# The nanoscale phase distinguishing of PCL-PB-PCL blended in epoxy resin by tapping mode atomic force microscopy

**DOI:** 10.1186/1556-276X-7-153

**Published:** 2012-02-23

**Authors:** Huiqin Li, Limin Sun, Guangxia Shen, Qi Liang

**Affiliations:** 1 Instrumental Analysis Center, Shanghai Jiao Tong University, Shanghai, 200240, China; 2 Research Institute of Micro/Nano Science and Technology, Shanghai Jiao Tong University, Shanghai, 200240, China

**Keywords:** tapping mode AFM, PCL-PB-PCL, phase image, force-probe

## Abstract

In this work, we investigated the bulk phase distinguishing of the poly(ε-caprolactone)-polybutadiene-poly(ε-caprolactone) (PCL-PB-PCL) triblock copolymer blended in epoxy resin by tapping mode atomic force microscopy (TM-AFM). We found that at a set-point amplitude ratio (*r*_sp_) less than or equal to 0.85, a clear phase contrast could be obtained using a probe with a force constant of 40 N/m. When *r*_sp _was decreased to 0.1 or less, the measured size of the PB-rich domain relatively shrank; however, the height images of the PB-rich domain would take reverse (translating from the original light to dark) at *r*_sp _= 0.85. Force-probe measurements were carried out on the phase-separated regions by TM-AFM. According to the phase shift angle vs. *r*_sp _curve, it could be concluded that the different force exerting on the epoxy matrix or on the PB-rich domain might result in the height and phase image reversion. Furthermore, the indentation depth vs. *r*_sp _plot showed that with large tapping force (lower *r*_sp_), the indentation depth for the PB-rich domain was nearly identical for the epoxy resin matrix.

## Introduction

Tapping mode atomic force microscopy (TM-AFM) has become a widely used technique to study the structures and properties of heterogeneous polymers at nanometer scale [1-9]. In a TM-AFM measurement, a cantilever is forced to oscillate with the probe tip at a given amplitude (*A*_0_). Then, the cantilever is brought close to the specimen and made to tap the surface with a given reduced set-point amplitude (*A*_sp_). The probe-sample interaction can introduce a phase shift in the vibration with respect to that of *A*_0_. So, TM-AFM measurement can obtain both height and phase images simultaneously. Height image can reflect the topographical and morphological structures, while phase images are sensitive to the physical and chemical properties of the studied material, such as stiffness, viscoelasticity, and chemical composition [3,10-12].

However, the contrasts of the height and phase images sensitively depend on experimental conditions [1,13-16] including cantilever force constant, tip shape, free amplitude *A*_0_, and set-point amplitude ratio (*r*_sp_) (equals to *A*_sp_/*A*_0_). This leads to difficulties in image interpretation for heterogeneous polymer samples. Thus, interpretation of the images has attracted considerable attention. To understand the results, a force-probe mode is performed on different materials [6,13,17]. In this mode, the amplitude and phase shift of the tapping cantilever are measured as a function of the varied tip-sample distance in order to study the indentation response of polymer surfaces in nanoscale. The tip penetration into the compliant sample is large, while very little penetration occurred on the stiff sample [18,19]. If the matrix is more compliant compared with the domain regions or the domain size is much larger than the probe tip diameter (about 20 nm), the indentation difference is not affected by the tip size [1,2,6,19]. In most of the reports, the investigated polymer samples were prepared by solution casting, so enrichment which occurred at the sample surfaces would affect the results except for the scan parameters [4,6,20,21]. In this paper, we studied a new heterogeneous polymer which has a more compliant domain region with the size of about 20 nm by TM-AFM. The experiments were performed on the ultrathin section of the polymer to study the structure of the bulk material. To interpret the results, we carried out the force-probe measurement on the microdomains and the matrix.

**Figure 1:**
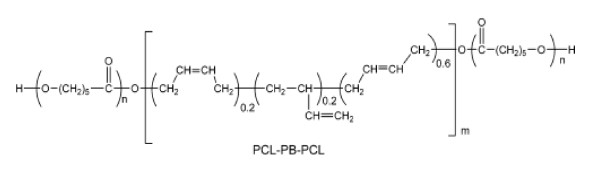
**Molecular structure of PCL-PB-PCL triblock copolymer**.

**Figure 2:**
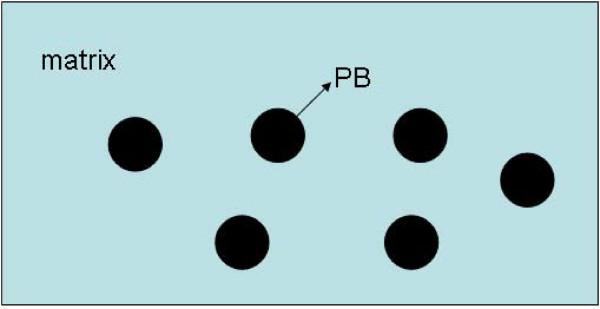
**Schematic nanostructure of the blend of PCL-PB-PCL triblock copolymer and epoxy resin**.

In this research, the polymer blends of epoxy resin with amphiphilic poly(ε-caprolactone)-polybutadiene-poly(ε-caprolactone) (PCL-PB-PCL) triblock copolymer (chemical structure seen in Figure 1) was studied by TM-AFM as in our previous work [22] which indicated that the nanostructures in the blend were formed due to the polymerization-induced microphase separation of PB subchains from the matrix, cross-linked epoxy networks, whereas the PCL remained mixed with the matrix (nanostructure seen in Figure 2). To identify the components of PCL-PB-PCL within the cross-linked epoxy resin, we carried out TM-AFM studies on the polymer blend. To help analyze the height and phase images, we performed transmission electron microscopy (TEM) measurement and force-probe measurement on the polymer blend. Our work demonstrated that to obtain true height and phase images of heterogeneous polymers and avoid artifact interference, it is important to select appropriate measurement parameters and suitable tips. Additionally, the tip indentation should be considered as well.

## Experiments

### Sample preparation

Triblock copolymer of PCL-PB-PCL was synthesized, and the blend system of PCL-PB-PCL and epoxy resin was prepared according to the procedure reported in our previous work [22]. The molecular weight of the PCL-PB-PCL was determined by hydrogen-1 nuclear magnetic resonance (^1^H NMR). It was found that *M*_n _is 14,600 and the weight fraction of PB subchains in the PCL-PB-PCL is 35%. According to ^1^H NMR results, the content of PCL-PB-PCL in the polymer blends is about 10% by weight. The specimen section of the polymer blends was prepared using a microtome machine (*ca*. 70 nm in thickness) and used for TEM and TM-AFM examination.

### Characterization techniques

The TEM measurement was performed with a high-resolution TEM instrument (JEM-2010, JEOL, Tokyo, Japan) at an acceleration voltage of 120 kV. The samples were stained with OsO_4 _to improve the image contrast. The stained specimen section was placed in a 200 mesh copper grid for observation.

TM-AFM measurements were performed with an AFM instrument (Nanoscope V, Veeco, Plainview, NY, USA) at ambient condition. The commercial Si cantilevers with two types of force constant (3 and 40 N/m) were used for tapping mode measurements. The height and phase images were recorded simultaneously using the AFM instrument. Tapping mode images were taken at the fundamental resonance frequency of the Si cantilever with an ultra-sharp tip (curvature radius approximately 10 nm). The typical scanning speed was 1 Hz for all measurements.

## Results

### Structure of PCL-PB-PCL-blended epoxy resin film

**Figure 3:**
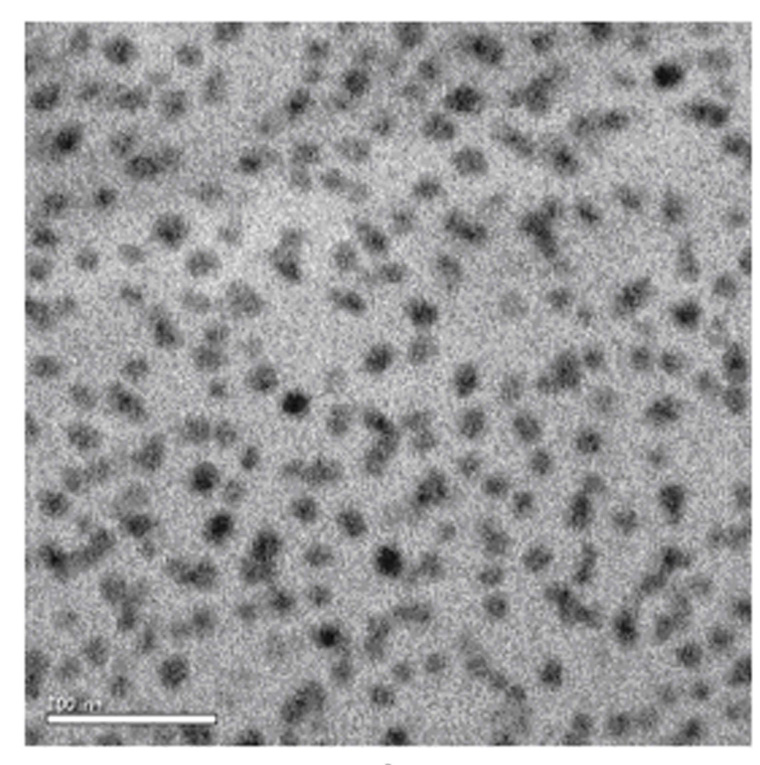
**The TEM image of the PCL-PB-PCL-blended epoxy resin film**.

To unambiguously identify the components of the blends, TEM image was taken. Figure 3 shows the TEM image obtained for the ultrathin section of the PCL-PB-PCL-blended epoxy resin sample. Since the PB domains are preferentially stained with OsO_4 _due to the C = C double bonds, the dark regions are assigned to PB microphases, whereas the bright regions are assigned to epoxy matrix and PCL because of no stain effect for these two components [13,22,23]. Figure 3 shows that PB forms nanostructures in the system and its size is about 20 nm.

### Effect of cantilever stiffness on AFM image contrast

**Figure 4:**
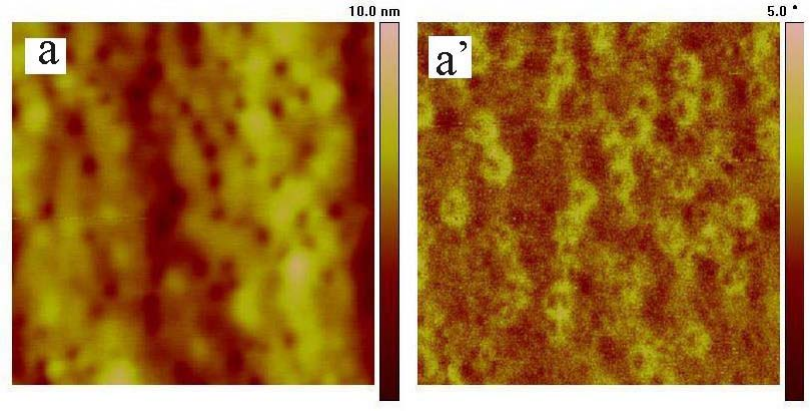
**Height (a) and phase (a') images of the blended film**. Taken with a softer tip cantilever with a spring constant of 3 N/m at *A*_0 _= 60 nm and *r*_sp _= 0.5. The scanning size is 500 nm × 500 nm.

TM-AFM images are affected by cantilever stiffness dramatically [17,24]. In this research, a weaker cantilever with a force constant of 3 N/m was used. A series of phase and height images were obtained at *A*_0 _= 60 nm with *r*_sp _which varied from 0.1 to 0.95. Figure 4 shows that no clear contrast was observed in the phase and height images obtained at *r*_sp _= 0.5. Since the other images have similar contrast to Figure 4, they are not presented here.

Under ambient condition, the contamination layer mainly composed of water is presented on the sample surface and leads to an attractive interaction between the cantilever and the surface water layer by capillary force [1]. A weaker cantilever with smaller amplitude is easy to be controlled by this attractive force. So, in order to get clear contrast for a phase image, a stiffer cantilever and a larger amplitude should be used.

### Effect of *r*_sp _on AFM image contrast

It was reported that the relative image contrast of chemically different regions depended sensitively on *r*_sp _and probe tips [1,14,24,25]. According to these works, in our research, a series of height and phase images were taken at different *r*_sp _(ranging from 0.95 to 0.1) using a stiffer tip with a force constant of 40 N/m.

**Figure 5:**
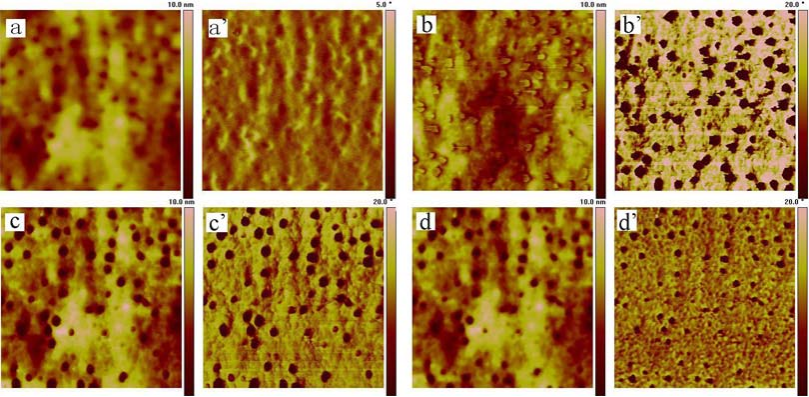
**Height (a-d) and phase (a'-d') images of the blended film at ***A*_0 _**= 30 nm**. (a, a'): *r*_sp _= 0.95; (b, b'): *r*_sp _= 0.85; (c, c'): *r*_sp _= 0.77; (d, d'): *r*_sp _= 0.1. The spring constant is 40 N/m, and the scan size is 500 nm × 500 nm.

Figure 5 shows a series of TM-AFM height (a, b, c, d) and phase (a', b', c', d') images recorded at 30 nm of the *A*_0 _and at different *r*_sp _of 0.95 (a, a'), 0.85 (b, b'), 0.77 (c, c'), and 0.1 (d, d'). It was found that the height and phase image contrasts were varied with the change of *r*_sp_. At weak tapping with the *r*_sp _of 0.95, the image contrast differences between PB-rich and epoxy matrix are not clear both in the height (a) and phase (a') images. However, decreasing the *r*_sp _value to 0.85, the image contrasts are dramatically increased (b, b', c, c'). At harder tapping with the *r*_sp _of 0.1, the size of the nanostructure measured from the height (d) and phase (d') images was 25% smaller compared with that from the images taken at the *r*_sp _of 0.77. An abnormal phenomenon, which was observed at *r*_sp _= 0.85, was that the height (c) image contrast was completely reverse relative to any other height images, though the phase (c') image contrast looked similar to that recorded at *r*_sp _= 0.77. The variations of the contrast of height and phase images at different *r*_sp _were different from previous results [4,7,26,27].

### Force-probe measurement on different domains

**Figure 6:**
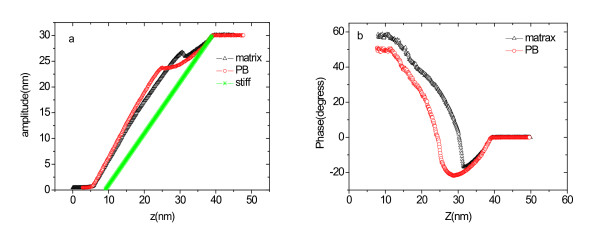
**Amplitude (a) and phase shift (b) as a function of ***z ***for matrix and PB regions**. The spring constant is 40 N/m.

**Figure 7:**
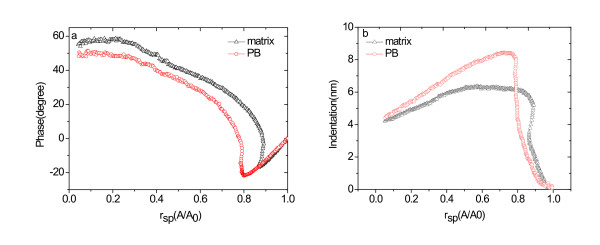
**Phase shift (a) and indentation (b) as a function of ***r*_sp _**for matrix and PB regions**. The spring constant is 40 N/m.

As observed in the 'Effect of *r*_sp _on AFM image contrast' section, the contrast of height and phase images depended sensitively on both measurement conditions and tip-sample interaction. To investigate the factors affecting the image contrast further, force-probe measurements were performed on the nanostructure domain and the matrix region of the sample surfaces (Figures 6 and 7).

Figure 6a shows the amplitude-*z *parameter vs. *A*(*z*) function curve obtained at *A*_0 _= 30 nm for the PCL-PB-PCL and epoxy resin blend system. In Figure 6a, the solid line represents the values of the amplitude *A*_is_(*z*) for an infinitely stiff sample assuming a zero indentation depth (*δ*). The *A*(*z*) curves observed for the PB and epoxy resin blend system lie above the *A*_is_(*z*) curve because of the indentation on these samples. Since the deformation of the stiff silicon tip can be negligible compared with the sample indentation, the indentation depth *δ*(*z*) at a given *z *could be estimated from the equation *δ*(*z*) = *A*(*z*)-*A*_is_(*z*) [13,18]. The *δ*(*r*_sp_) curves were also obtained in a similar way. The *δ*(*r*_sp_) curves were plotted in Figure 7b. Finally, the phase shift-*z *distance curve and the phase shift-*r*_sp _curve were deduced from the amplitude-*z *and the phase angle-*z *curves, which are shown in Figures 6b and 6a, respectively.

## Discussion

It was found that the 'dark circle' shown in the height image and the phase image (Figure 5c, c') was close to the nanopores shown in the TEM image. So, the morphology and the phase separation observed in TM-AFM images could be verified.

The phase image contrast revealed in Figure 5 can be interpreted according to the phase shift-*r*_sp _curve. For *r*_sp _> 0.85, the phase shift is negative both on PB and matrix, and the phase shift difference between PB and matrix is small, which accounts for the obscure phase contrast obtained at *r*_sp _= 0.95 (shown in Figure 5a'). As *r*_sp _is in the region of 0.77 to 0.85, the phase shift becomes larger on the matrix than on PB, and the largest phase shift difference between PB and matrix was observed. So, in the phase images obtained at *r*_sp _= 0.77 (Figure 5c') and at *r*_sp _= 0.85 (Figure 5b'), the PB-rich region shows to be darker than the matrix region, and the phase images show maximum contrast. While the *r*_sp _decreases to 0.1 (Figure 5d'), a clear phase contrast is also obtained, and the PB-rich region remains to have a dark contrast. Additionally, it was found that the size of PB-rich domains decreases compared with that shown in Figure 5b', c' in both height and phase images. In the blend, the cross-linked epoxy resin of the matrix is a thermoset polymer, and the PB polymer of the domain materials is liquid at room temperature [22]. The two materials are quite different in stiffness. So, it is necessary to take the indentation depth of the probe tip into consideration. The indentation depth-*r*_sp _curve (Figure 7b) reveals that the indentation depth of the PB-rich region is larger than that of the matrix when *r*_sp _is more than 0.8, and it is reverse when *r*_sp _is less than 0.8. For larger *r*_sp_, the tip-sample interaction on the matrix of PCL mixed with epoxy will be more affected by the attractive capillary force than on the PB domains because the matrix of epoxy resin is more hydrophilic than the PB domain due to the oxygen atoms. So, the stiffer matrix shows larger indentation depth. For smaller *r*_sp_, the indentation depth is nearly identical for the PB-rich domain and for the matrix, and the indentation depth is just about several nanometers. Considering that the radius size of the PB domain (approximately 10 nm) is close to the tip curvature radius (approximately 10 nm), when *r*_sp _is dramatically decreased, the tip contacts both the PB domain and the matrix simultaneously. The stiff edge of the domain will prevent the tip from indenting into the compliant PB-rich region. So, the size of the domain obtained is just the size of the tip, which results in the shrinkage of the measured size of the PB domain.

Many studies showed that to obtain images which describe the 'true' topography of a sample surface, the images should be recorded using sufficiently high set-point amplitudes and high *r*_sp _values. In this study, at *r*_sp _= 0.95 to 1.0, significant tip indentation in the PB region and the matrix did not take place (see Figure 7b). We thought that at this condition, the true topography is obtained (Figure 5a), but at *r*_sp _= 0.85, the height image contrast shown in Figure 5b was completely reverse. We found that in the phase shift-*r*_sp _curve, at *r*_sp _= 0.85, the interaction force between the tip and the sample varied from the attractive interaction on the PB into the repulsive interaction on the matrix (Figure 7a), leading to a large decrease in amplitude. Thus, the PB region looks higher than the real topography in the height image as far as the feedback mechanism is concerned. So, a height artifact is observed in Figure 5b, instead of the height image contrast inversion. While *r*_sp _≤ 0.77, the tip-sample interaction force is repulsive for both the PB and the matrix, so the height images did not undergo reverse but influenced by the effect of tip indentation.

## Conclusion

The above results demonstrated that it is important to select appropriate scanning parameters during TM-AFM measurement in order to get a good image with the clear morphology and topography information. The topography and nanoscale phase separation of PCL-PB-PCL-blended epoxy resin have been studied by tapping mode AFM at different *r*_sp _values and with different tips. The stiffer tips are found to be desired to get clear phase shift images. According to the force-probe measurements, the optimum condition for TM-AFM measurement is 0.77 ≤ *r*_sp _< 0.85. At lower *r*_sp_, the size of compliant PB-rich domains decreases because the tip size is close to the dimension of PB-rich domains. At *r*_sp _= 0.85, the tip-sample interaction force varies from the attractive interaction on the PB into the repulsive interaction on the matrix, which leads to the height image reverse phenomena.

For the reasonable interpretation of the TM-AFM phase and height images, it is necessary to take the indentation depth and the tip-sample interaction force into consideration. The present study is general and should be easily transferred for the analysis of a similar system.

## Abbreviations

*A*_0_: given amplitude; *A*_sp_: set-point amplitude; PCL-PB-PCL: poly(ε-caprolactone)-polybutadiene-poly(ε-caprolactone); *r*_sp_: set-point amplitude ratio; TEM: transmission electron microscopy; TM-AFM: tapping mode atomic force microscopy.

## Competing interests

The authors declare that they have no competing interests.

## Authors' contributions

HL carried out the AFM measurements and drafted the manuscript. LS and GS edited the manuscript. QL participated in the analysis and guidance of the study. All authors read and approved the final manuscript.
